# What Goes Around: the process of building a community-based harm reduction research project

**DOI:** 10.1186/s12954-017-0199-1

**Published:** 2017-11-16

**Authors:** Chelsea Jalloh, Shohan Illsley, John Wylie, Paula Migliardi, Ethan West, Debbie Stewart, Javier Mignone

**Affiliations:** 1 The 595 (Manitoba Harm Reduction Network), Winnipeg, Canada; 2 Faculty of Education, University of Manitoba, Winnipeg, Canada; 3 Sexuality Education Resource Centre, Winnipeg, Canada; 4 Department of Medical Microbiology, University of Manitoba, Winnipeg, Canada; 5 Department of Community Health Sciences, University of Manitoba, Winnipeg, Canada

## Abstract

**Background:**

Often, research takes place on underserved populations rather than with underserved populations. This approach can further isolate and stigmatize groups that are already made marginalized. What Goes Around is a community-based research project that was led by community members themselves (Peers).

**Case presentation:**

This research aimed to implement a community-based research methodology grounded in the leadership and growing research capacity of community researchers and to investigate a topic which community members identified as important and meaningful. Chosen by community members, this project explored how safer sex and safer drug use information is shared informally among Peers. Seventeen community members actively engaged as both community researchers and research participants throughout all facets of the project: inception, implementation, analysis, and dissemination of results. Effective collaboration between community researchers, a community organization, and academics facilitated a research process in which community members actively guided the project from beginning to end.

**Conclusions:**

The methods used in What Goes Around demonstrated that it is not only possible, but advantageous, to draw from community members’ involvement and direction in all stages of a community-based research project. This is particularly important when working with a historically underserved population. Purposeful and regular communication among collaborators, ongoing capacity building, and a commitment to respect the experience and expertise of community members were essential to the project’s success. This project demonstrated that community members are highly invested in both informally sharing information about safer sex and safer drug use and taking leadership roles in directing research that prioritizes harm reduction in their communities.

## Background

“Nothing about us without us” is a popular sentiment within the field of HIV/AIDS [1]. More than a slogan, this statement is a guiding principle for working at a community level; a call for a commitment that policies and programs should be developed with the full participation of the group(s) affected by them. This includes the participation of individuals who are often excluded from political, social, and economic opportunities, such as individuals who experience poverty, individuals involved in sex work, and individuals who use substances. In addition to policy and program development, the statement is applicable to research—particularly research that involves underserved groups. Within the field of healthcare and health research, groups who experience health disparities may be referred to as “marginalized,” “vulnerable,” or “underserved” [2]. While these terms are often used interchangeably, there are notable distinctions [2]. Other scholars have used the term “vulnerable” populations, which we believe is an inaccurate, increasingly broad [3], and potentially discriminatory description of the abovementioned groups. Throughout this paper, the description of “underserved” relates to individuals who, as a result of their membership in a particular group, may experience difficulties in accessing and obtaining healthcare, may receive a lesser quality of both healthcare and interpersonal treatment, and/or may receive treatment that does not sufficiently address their needs [4].

The process of how to put the statement “Nothing about us without us” into action is unique in each particular context. How best to identify and capitalize on strengths and opportunities, navigate challenges, and build reciprocal collaborative relationships among stakeholders are just some of the key considerations. The research project described here, “What Goes Around: How Peers Use Their Social Networks to Share STBBI Education and Information” (*What Goes Around*), is an exemplar of how community members can effectively engage with, and lead, a multi-faceted community-based research (CBR) project.

At the time this project began (2011), in the province of Manitoba, Canada, rates of new HIV infections were increasing [5]. While rates of new HIV infections have stabilized in Canada since 2002, over the course of 2010, the rate of new HIV cases in Manitoba (12.1 per 100,000) remained higher than the national average (8.2 per 100,000) [6]. In 2011, leading modes of HIV transmission in Manitoba were heterosexual sex (67%), followed by men who have sex with men (18%), and injection drug use (11%) [5]. Of note, recently—and for the first time since data collection began in 2007—leading modes of transmission in Manitoba have shifted to men who have sex with men (39%), heterosexual sex (33%), HIV-endemic country (21%), and injection drug use (8%) [7]. Over-representation of Indigenous peoples remains an important issue in 2015 with 23% of new clients to the Manitoba HIV program self-identified as Indigenous (First Nations, Metis, or Inuit) [7]. At present, Manitoba has one of the largest proportions of women, compared to men, living with HIV in Canada [7].

The 595 Prevention Team (formerly The Manitoba Harm Reduction Network) is a direct response to the rates of HIV within Manitoba. The 595 is a province-wide network of over 100 member organizations working to prevent the transmission of sexually transmitted and blood-borne infections (STBBIs), particularly HIV and hepatitis C (HCV) throughout Manitoba. The 595 is not a service delivery organization; rather, The 595 brings together organizations, policy makers, academics, and community leaders to make recommendations regarding the development, implementation, and evaluation of initiatives rooted in the principles of harm reduction.

The heart of The 595 is a group of up to 20 individuals who comprise The 595 Peer Working Group (Working Group). The members of the Working Group, the “Peers,” are people who are living with or affected by HIV and/or HCV and who are actively involved in preventing the transmission of STBBIs in their communities through harm reduction. Working as the expert advisory body of The 595, the Working Group informs the organization on issues related to safer drug use, safer sex practices, harm reduction strategies, peer engagement strategies and tools, program development and implementation, knowledge translation, and support for individuals affected by STBBIs, including HIV and HCV. The Working Group includes women, men, transgender, and pangender individuals with a diverse range of experiences such as sex work, substance use, and homelessness. Members of the Working Group range in age from their mid-30s to mid-60s; the majority of Peers self-identify as Indigenous. At the time of this project, based on self-reported data, all 17 members of the Working Group indicated that they had used, or currently use, substances and alcohol. Of the 11 Working Group members who reported current substance use, substances of choice included marijuana, crack, cocaine, opiates, solvents, and alcohol. Close to 90% of the members of the Working Group indicated that they had injected drugs at some point; other methods of substance use included snorting, smoking, and swallowing drugs.

In contrast to the spirit of “Nothing about us without us,” The 595 acknowledged that much of the community-level research regarding the prevention of HIV and STBBIs was being conducted *on* community members, not *with* community members. Eager to change this practice, the Working Group explored conducting CBR themselves. This resulted in the completion of two CBR projects: *Hell Yeah I'm an Expert!*: *A snapshot of peer engagement in HIV/AIDS, STI’s and BBP prevention initiatives* (2008-2009) and  *DIY distribution: Peer directed harmreduction supply distribution!* (2009-2010) [8]. As a result of participating in these CBR projects, Peers increased their capacity to initiate, conduct, and direct research.

In 2013, the Working Group took steps to undertake an innovative new CBR project: *What Goes Around*. Drawing upon the existing skills and capacity of the Working Group as community researchers, and with the support from faculty members at the University of Manitoba, the Working Group set out to implement a CBR process that was engaging, meaningful, and participatory for all researchers involved.

## Case presentation

### Project objectives

The objective of the study, as decided by the Peers, was to explore the many ways in which they share harm reduction information and supplies within their personal networks. From the beginning, *What Goes Around* was designed with specific methodological objectives in mind. A primary goal was to assemble a team of academic researchers to support the leadership and creativity of the Working Group in all aspects of the study. The participating academic researchers were faculty members from various departments of the University of Manitoba, including Community Health Sciences and Medical Microbiology. These particular faculty members were selected due to their extensive experience conducting CBR with underserved groups both nationally and internationally and also because of their strong backgrounds in STBBI prevention and research. Several principles guided the Peers’ and faculty’s involvement in the CBR process:

1. To collaboratively engage community members and academic researchers equally
2. To encourage a reciprocal learning and fully participatory process for team members
3. To focus on local community capacity building
4. To strive for an empowering process through which community participants increase control over their lives
5. To achieve a balance between research and action [9]

### Project activities

Apart from the specific research objectives, the overarching process goal of *What Goes Around* sought to authentically engage members of the Working Group in the development and direction of a CBR project. This process was iterative, and the Working Group was involved specifically in the key project activities detailed below.

#### Developed CBR project topic

At regular Working Group meetings (approximately every 8 weeks), Peers engaged in discussions about their findings from previous CBR projects, what topics invited further exploration, and how a new CBR project could build upon their previous research. A private group on Facebook was the primary method to share CBR-related information with the Working Group (one Peer without Facebook was contacted via phone).

#### Developed guiding research questions

Drawing from topics of interest from the two previous CBR projects, and in dialog between the Working Group and The 595 executive director, the research objectives of *What Goes Around* were to:

1. Explore how the Working Group shares safer drug use and safer sex information informally within their own social networks
2. Explore how the Working Group seeks to reduce the spread of STBBIs in their community
3. Explore how the Working Group provides care and support to those who are HIV and/or HCV positive

#### Increased CBR capacity

Several members of the Working Group attended a Summer Institute on Community Based Research at the University of Regina (June, 2011). Upon their return to Winnipeg, institute attendees shared the knowledge they gained with the wider Working Group and served as CBR resources throughout the project.

#### Assisted in obtaining research funding

The Working Group created a CBR Committee that included two representatives from Working Group, the executive director of The 595 and three academics from the University of Manitoba and a community organization. The larger Working Group collaborated with members of the CBR Committee to compose a successful proposal for a Catalyst Grant from the Canadian Institutes of Health Research (CIHR). Proposal preparation involved developing specific research objectives, discussing the benefits and limitations of different data collection and analysis methodologies, and identifying project logistics (including budget and project management processes). Following the successful acquisition of CIHR funding, the CBR Committee oversaw the research project and worked in concert with the larger Working Group during project implementation. Part of the funding was used to hire a research coordinator to provide support to the project and to liaise between the CBR Committee and the Working Group. The interviews to select the coordinator were conducted by the executive director of The 595 and two representatives from the Working Group.

#### Development of research processes and tools

Because *What Goes Around* sought to explore how Peers share harm reduction information within their social networks, members of the Working Group themselves served as the data sources for the research. The executive director of The 595 was the primary interviewer. However, in recognition of the inherent power dynamics in the interaction between the executive director and Peers, the Working Group developed multiple options for data collection. Each Working Group member could choose to be interviewed solely by the executive director, to be interviewed by the executive director and the research coordinator together, to be interviewed by the research coordinator, or to decline to be interviewed.

The CBR committee and the Working Group collaborated to develop a mixed-methods approach for data collection. At meetings specifically about *What Goes Around* (every 2–4 weeks), the whole Working Group discussed the project and shared their feedback and perspectives. The Working Group’s input was communicated to the CBR Committee via the two Peer representatives on the committee and the research coordinator. The input of the Working Group was essential in order to craft the questionnaire and interview guide with language that they preferred and to reflect the subject areas they identified as most important.

Members of the Working Group who chose to participate in data collection (*n* = 17) initially completed a short quantitative questionnaire to provide demographic information and to outline how they receive and share safer sex/safer drug use information and supplies. All descriptive categories on the questionnaire were chosen by the Working Group. This was particularly important for questions pertaining to gender, sexuality, health status, and ethnicity. The questionnaire was followed by a verbal semi-structured interview, administered by the interviewer(s) of the participant’s choosing. The interviews were audio recorded and transcribed verbatim.

In addition to the development of the data collection methodology, the Working Group’s contributions to the development of an informed consent process were critical. Members of the Working Group have frequently experienced having research done on them. Consequently, participation in the development of the consent process allowed the Working Group to create a consent document that was comprehensive, understandable, and that clearly explained the risks and benefits of research participation. A first draft of the consent form was presented to the Working Group for feedback. The group revised the consent form by simplifying the language, clarifying the issues of honoraria and bus tickets, and including relevant support phone numbers (in the event that a participant required additional support post-interview).

#### Assisted in composing ethics board submission

Prior to data collection, the Working Group assisted the CBR Committee in writing an ethics submission that received approval from the Joint Faculty Research Ethics Board at the University of Manitoba.

#### Participated in data collection as “interviewees”

At the time of data collection, the Working Group consisted of 17 members, all of whom chose to participate in an interview (December 2012–February 2013). At the time of the interview, a written copy of the consent form was provided to the Peer and the information was also reviewed orally by the interviewer(s). It was made clear that Peers were free to cease participation at any time during the interview without penalty. Following the interview, each participant received $20 honoraria and two bus tickets.

#### Capacity building: data analysis and interpretation

Building on skills and knowledge developed from previous CBR experience, the Working Group engaged in on-going research capacity-building activities. As an introduction to the process of data sorting, the research coordinator provided the Working Group with a grocery list of ten items. In small groups, Working Group members “sorted” the grocery list—each small group decided how they would sort the list (e.g., by food groups, color of food, aisle numbers in the grocery store). Following the sorting exercise, the whole group discussed how each of the small groups had sorted their list and also brainstormed what additional criteria for sorting could be used (e.g., items that are full priced vs. items on sale, local vs. imported food). Peers talked about how they might sort items that could fall into multiple categories (such as pizza) and discussed how people conducting data sorting and analysis must negotiate and explain their data sorting choices. Furthermore, the group discussed the importance of identifying which question(s) the study was trying to answer in order to best choose how to organize the data to respond to that question.

Next, the Working Group members applied their data sorting skills to an actual interview transcript (all identifiers removed). Again working in small groups, they were provided with two pages of an interview transcript and asked to highlight any information that answered the question “What safer sex or safer drug use information does this person share?” This exercise was quite a bit more challenging than the grocery sorting exercise. One of the notable challenges was Peers spending significant time evaluating the legitimacy of the information the interviewee shared. This development provided an opportunity for the research coordinator to clarify that, in this context, the data sorting process does not judge if the data is “right” or “wrong”; rather, it groups similar ideas together to try to create themes/commonalities in order to answer a specific question.

Upon completion of this second exercise, the general consensus was that members of the Working Group were not interested in participating in the “nitty gritty” of data sorting and analysis. However, the Working Group expressed that they had gained an understanding of the data sorting and analysis processes that would be used in *What Goes Around* and wanted to participate in the data verification process and member checking [10].

#### Ongoing data analysis and verification

Ongoing data verification was imperative to ensure accuracy of findings. Following the direction of the Working Group, the executive director of The 595 and the research coordinator sorted and coded the project data. Throughout the data analysis process, they routinely met with the Working Group to share initial and emergent findings, to check that preliminary interpretations of data were “on the right track,” and to ask about items that required further information. This process was particularly useful to clarify the Working Group’s definitions of some of the terms that came up during the interviews, such as their working definition of “mental health,” and slang terms associated with substance use.

Due to the sources of data being the members of the Working Group themselves, in two instances, specific quotes were modified to be included in the data analysis. In these cases, while pseudonyms were used, it could still be possible for individuals familiar with the Working Group members’ experiences to identify the source of the statement by the specific information shared (e.g., reference to a particular physical characteristic, reference to a particular life event). The information shared in these two quotes was paraphrased to exclude identifying information and included in the analysis. However, how to effectively protect the identity of specific interview participants when the sources of data are also involved in data analysis and verification is an important consideration.

#### Knowledge dissemination plan

**Fig. 1:**
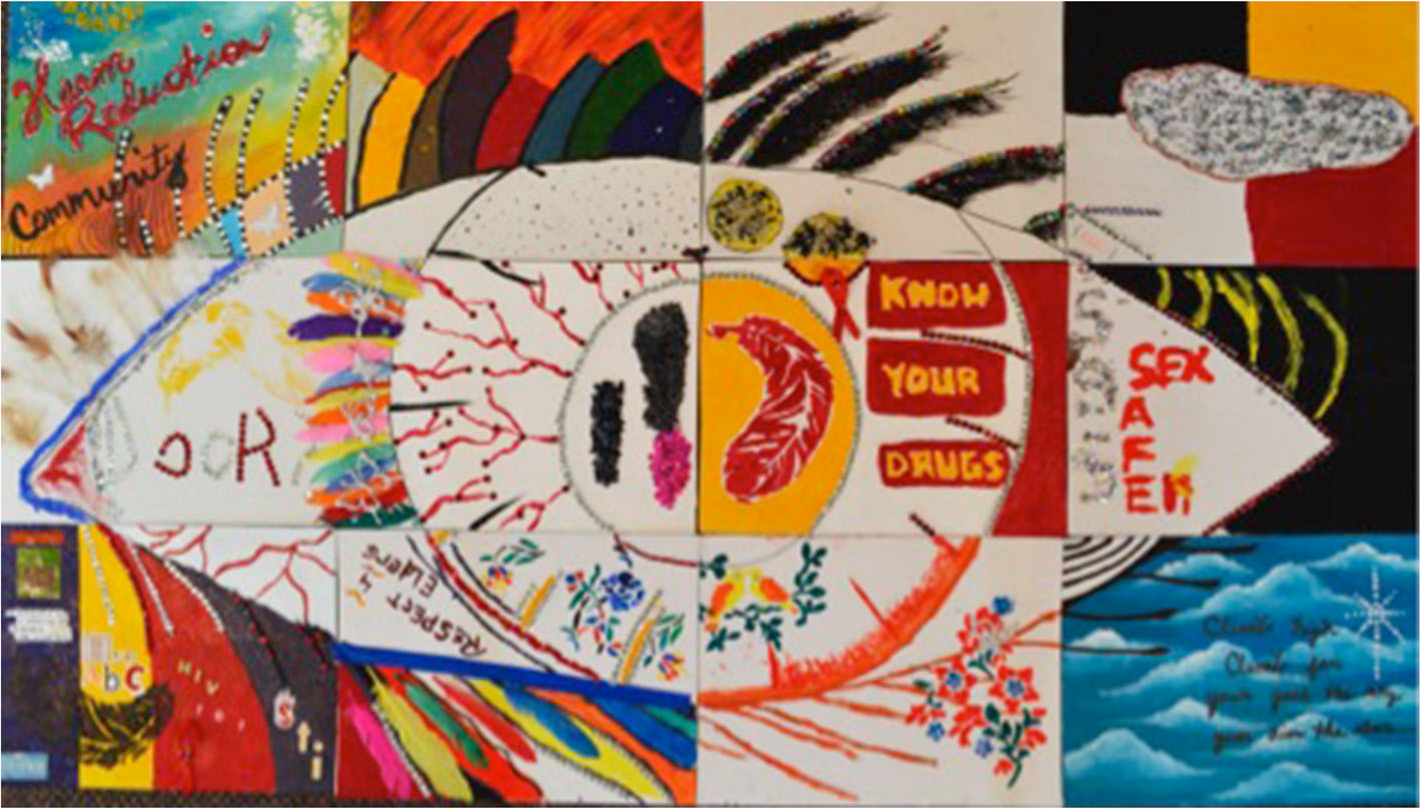
A vision board created by the Peers to represent their experience throughout the CBR project

**Fig. 2:**
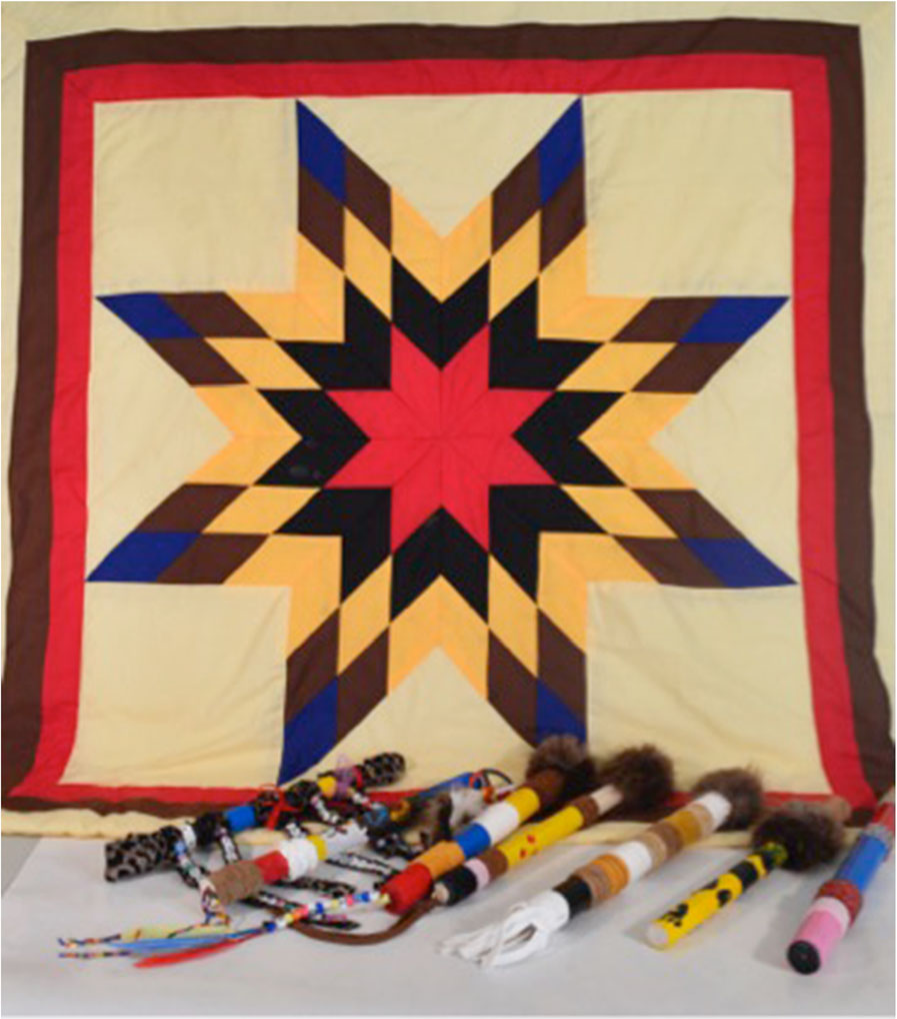
Each Peer created a personalized talking stick as a symbol of their participation in the CBR

At the conclusion of the data analysis, the Working Group identified who they thought would benefit from receiving information about the findings of their research. The list of potential beneficiaries was robust and varied, from service providers, to community members, to media, and to family and friends. The Working Group also determined what forms of knowledge dissemination would be the best fit for the different audiences. Knowledge dissemination strategies included:

i. *Print materials (Peer handout)*—for more formalized and/or academic conferences, the two Working Group representatives on the CBR committee worked with the research coordinator to create a handout for community members. This Peer handout was complemented by an additional handout produced specifically for service providers [11]. The Peer handout emphasized the results of the study that the Working Group identified as the most important and communicated the information in language that was deemed as accessible and appropriate by the Working Group representatives.
ii. *Vision board—*working with a talented Anishinabe artist, the Working Group created a vision board to represent their experiences coordinating and participating in *What Goes Around*. Each member of the Working Group received a canvas rectangle to decorate independently, which would eventually be assembled with all rectangles into a large image of an eye; a symbol that was chosen by the group. The individual rectangle would represent a component of each individual’s involvement in the CBR that they found personally meaningful, and the image of an eye representing the work of the group (Fig. 1).
iii. *Talking sticks*—the Working Group expressed interest in having an Indigenous elder come to share a teaching and lead the group in creating talking sticks. The use of talking sticks is practiced by many Indigenous peoples in North America to provide an opportunity for individuals to have the space and time to share their thoughts during a gathering. As members of the Working Group explained, talking sticks were a fitting symbol because the Peers act as “talking sticks” in their communities when they share information about safer sex and safer drug use. Each Peer created an individual talking stick to keep as a reminder of their participation in the project (Fig. 2).
iv. *Public presentations*—a specific presentation of research findings for service providers who work in the fields of health and social services took place several months after the completion of the study (2013). During this event, a number of Working Group members shared their experiences of participating in the development and execution of *What Goes Around*. A subsequent community forum to share the study findings with other community members was also organized in 2014. Poster and oral presentations outlining *What Goes Around* project processes and findings were presented nationally at the Prairie HIV Conference (2013) and the Canadian Association of HIV Research Conference (2014).

### Impact and moving forward

In addition to the success of obtaining and sharing pertinent study findings of *What Goes Around*, the successful implementation of the research process itself has acted as a springboard for a number of innovative initiatives on a larger scale.

The Working Group is well-established as a strong resource to advocate for individuals living with/affected by HIV within the urban boundaries of Winnipeg. However, rural, remote, and Northern communities in Manitoba face critical challenges related to HIV. These challenges reflect barriers faced by communities outside of urban centers all over Canada [12]. In fact, while all HIV-specific care services are located within Winnipeg, in the last decade, between 20 and 30% of incident HIV cases in Manitoba have consistently been reported in communities that are outside of Winnipeg, [7]. CBR initiatives similar to the research undertaken by the Working Group in Winnipeg could have similar value in terms of empowering people affected by HIV to identify their own research agendas, priorities, and processes.

To directly support rural, remote, and Northern communities undertaking their own research, a 2-day CBR capacity-building event was held in 2015 in Winnipeg. This event, “Remote Control,” was funded by a successful CIHR Planning Grant and the Aboriginal HIV & AIDS Community Based Research Collaborative Centre. Twenty-five stakeholders from throughout the province attended the event, including representatives from local and national community-based organizations, research and academic centers, and rural, remote, and Northern communities. Through conversation and activities, the objectives of the event were to identify participants’ local challenges related to HIV care and support, identify local research priorities, and build capacity for CBR in rural, remote, and Northern Manitoba [13]. Furthermore, the success of the *Remote Control* event facilitated the creation of the development of a Rural, Remote and Northern Manitoba HIV CBR Network. This network collaboratively submitted a CIHR Operating Grant application. The application outlined three primary objectives as decided by the Rural, Remote and Northern Manitoba HIV CBR Network: (1) identify the care, treatment, and support needs of people living with HIV and affected communities in rural, remote, and Northern Manitoba; (2) strengthen partnerships among community members, community organizations, and the University of Manitoba and University College of the North; (3) increase and build on CBR capacity among community members, community organizations, and academic institutions.

The CIHR Operating Grant application was successful, and plans for project implementation are currently underway. Individual CBR projects will be undertaken in three rural, remote, and Northern communities: a First Nations community within Swampy Cree Tribal Council, Flin Flon, and Thompson, Manitoba. Drawing from the principles of CBR espoused in *What Goes Around*, each of these CBR projects will be guided by research teams comprised of community members, peer researchers, and academic researchers. Echoing the process of *What Goes Around*, central to this CBR approach is the importance of community members participating in every step of the research process (Kramer Diaz, Spears Johnson & Arcury, 2015) [14, 15]), and a fundamental appreciation of the knowledges and expertise of all individuals involved in the CBR initiative. The overarching goal of this next phase is to contribute to community members’ transformation and engagement as co-researchers on the team, not serving only as research participants and sources of data.

A number of lessons can be brought forward from the *What Goes Around* project into this expanded phase. One important consideration is the issue of timelines. In the case of *What Goes Around*, the Working Group dictated the timeline for the implementation of the project; this approach was often dynamic, flexible, and responsive to the lived experiences of the Peers (for instance, perhaps a Working Group meeting date got pushed back because several Peers had pressing family/personal/health matters and were unable to attend). At times, this posed a challenge for collaborators and academic partners (such as ethics review boards and funders) who are often used to more structured and rigid timelines. In addition to differing approaches to managing project timelines, collaborative processes which require information sharing with multiple stakeholders need more time than projects with a smaller number of collaborators involved. The involvement of multiple stakeholders from multiple backgrounds adds richness to the research process and results; however, the time that this richness requires is an important consideration in project planning. Project timelines should strive to incorporate flexibility and realistic and achievable expectations for the different groups involved. In addition, capacity-building activities proved to be highly important. Project timelines should also build in time for community researcher capacity building and knowledge exchange.

A second consideration is the importance of relationships [16, 17]. The value of an equitable partnership between community/peer researchers and academic researchers is clear. However, equally as important is that all members of a CBR team recognize the historical and contemporary power relationships that exist between the various team members [14]. There should be an emphasis on the importance of co-education (multi-directional) and active steps taken towards the rectification of knowledge imbalances [18, 19]. In the case of *What Goes Around*, the Working Group already had a well-developed, positive relationship with The 595 executive director. This relationship provided a solid foundation on which to build the collaborative relationships for the CBR project. For CBR projects where there is not an already existing relationship, particularly for groups that often experience systemic power disparities, time and purposeful effort need to be invested in order to grow the relationships that underpin the CBR project. Relationships continue to develop and change throughout the research process. In order to facilitate the development of positive collaborative relationships, it is imperative that each step of the research process be transparent and carefully explained to all stakeholders involved and questions that arise during the course of the research must be quickly and efficiently addressed.

## Conclusion

“Nothing about us without us!”—the *What Goes Around* project was an example of this phrase in action. The productive collaboration between the members of the Working Group, the executive director of The 595, the research coordinator, and the academic researchers in all aspects of this CBR project created a context in which all collaborators were able to benefit and learn from each other’s expertise and experiences. The positive momentum of *What Goes Around* resulted in the acquisition of funds to support other Manitoban communities affected by HIV to similarly undertake CBR projects that are directed by the community and that are for the community. The multi-dimensional collaborations between community members and academic researchers that will drive these future CBR projects provide opportunities for CBR research findings to directly inform the policies, programs, and practices that will better address the health experiences of people in Manitoba living with HIV.

## Abbreviations

CBR: Community-based research
CIHR: Canadian Institutes of Health Research
HCV: Hepatitis C
HIV/AIDS: Human immunodeficiency virus/acquired immune deficiency syndrome
STBBIs: Sexually transmitted and blood-borne infections
